# Korsakoff’s Syndrome and Alzheimer’s Disease—Commonalities and Specificities of Volumetric Brain Alterations within Papez Circuit

**DOI:** 10.3390/jcm12093147

**Published:** 2023-04-27

**Authors:** Shailendra Segobin, Melanie Ambler, Alice Laniepce, Hervé Platel, Gael Chételat, Mathilde Groussard, Anne-Lise Pitel

**Affiliations:** 1 Normandie Univ, UNICAEN, PSL Université Paris, EPHE, INSERM, U1077, CHU de Caen, Cyceron, Neuropsychologie et Imagerie de la Mémoire Humaine (NIMH), 14000 Caen, France; segobin@cyceron.fr (S.S.); alice.laniepce@univ-rouen.fr (A.L.); herve.platel@unicaen.fr (H.P.); 2 Normandie Univ, UNIROUEN, CRFDP (EA 7475), 76821 Rouen, France; 3 Normandie Univ, UNICAEN, INSERM, PhIND “Physiopathology and Imaging of Neurological Disorders”, Institut Blood and Brain @ Caen-Normandie, Cyceron, 14000 Caen, France

**Keywords:** Alzheimer disease, Korsakoff’s syndrome, thalamus, hippocampus, cingulate cortex, mammillary bodies, mammillothalamic tract, MRI

## Abstract

**Background**: Alzheimer’s disease (AD) and Korsakoff’s syndrome (KS) are two major neurocognitive disorders characterized by amnesia but AD is degenerative while KS is not. The objective is to compare regional volume deficits within the Papez circuit in AD and KS, considering AD progression. **Methods**: 18 KS patients, 40 AD patients (20 with Moderate AD (MAD) matched on global cognitive deficits with KS patients and 20 with Severe AD (SAD)), and 70 healthy controls underwent structural MRI. Volumes of the hippocampi, thalami, cingulate gyri, mammillary bodies (MB) and mammillothalamic tracts (MTT) were extracted. **Results**: For the cingulate gyri, and anterior thalamic nuclei, all patient groups were affected compared to controls but did not differ between each other. Smaller volumes were observed in all patient groups compared to controls in the mediodorsal thalamic nuclei and MB, but these regions were more severely damaged in KS than AD. MTT volumes were damaged in KS only. Hippocampi were affected in all patient groups but more severely in the SAD than in the KS and MAD. **Conclusions**: There are commonalities in the pattern of volume deficits in KS and AD within the Papez circuit with the anterior thalamic nuclei, cingulate cortex and hippocampus (in MAD only) being damaged to the same extent. The specificity of KS relies on the alteration of the MTT and the severity of the MB shrinkage. Further comparative studies including other imaging modalities and a neuropsychological assessment are required.

## 1. Introduction

Amnesia refers to severe episodic memory deficits that interfere with independent daily living. While amnesic cases have been described more than one century ago and have largely contributed to the understanding of memory function and substrates [1], pathophysiology of amnesia remains unclear. Amnesic patients are rare and their neuroimaging investigations even more. However, the study of brain abnormalities in amnesic patients from different etiologies makes inference possible regarding the brain mechanisms underlying amnesia and more generally concerning the substrates of episodic memory. 

We focused the present study on Alzheimer’s disease (AD) and Korsakoff’s syndrome (KS), which are two major neurocognitive disorders [2] resulting in amnesia associated with loss of autonomy in daily life. AD is a neurodegenerative disease that has historically been regarded as a medial temporal lobe amnesia, with the pathology centered on hippocampal atrophy at the early stage and progressively extending to neocortical areas [3]. Contrary to AD, KS has been studied as a model of diencephalic amnesia resulting most commonly from the combination of alcohol use disorder and thiamine deficiency, and characterized by brain abnormalities especially affecting the thalami and mammillary bodies [4,5]. Post-mortem and in vivo neuroimaging investigations highlighted the key role of the anterior thalamus in the pathophysiology of KS [6,7]. However, some literature reports thalamic abnormalities in AD [8] with neurodegeneration in the anterior thalami and a particular vulnerability of these nuclei in prodromal AD [9]. In the same vein, hippocampal volume deficits has been described in a group of five KS patients [10] and the severity of the memory impairment correlated with this hippocampal shrinkage. 

The regions crucial to both pathologies are actually part of a single brain circuit: the Papez circuit. There is increasing support in the literature to consider amnesia as the result of an impairment of a brain network responsible for memory, rather than to a particular region [9,11,12]. The anatomical differences and similarities between AD and KS patients can provide novel insights regarding the brain substrates of amnesia and more generally of episodic memory. From that perspective, structural abnormalities observed in AD and KS need to be directly compared, taking the neurodegenerative nature of AD and thus considering how AD progression could affect these comparison results with KS. 

The goal of the present study is thus to compare structural brain damage in these two amnesia which are hypothesized to result from different pathophysiological mechanisms primarily involving either the hippocampus (in AD), the thalamus (in KS), or the Papez circuit in general. The present investigation therefore focuses on evaluating the volumes of regions belonging to the Papez circuit and considered as hallmarks to each pathology, taking the potential effect of AD evolution into account.

## 2. Materials and Methods

### 2.1. Population

Forty patients with AD (20 with Moderate AD (MAD) and 20 with Severe AD (SAD)), 18 patients with KS, and 70 healthy controls (HC) were enrolled in this study. All groups were matched for gender and education but not for age (Table 1). All participants (and caregivers for patients when appropriate) provided written informed consent for inclusion in the study, which was approved by the local ethics committee of Caen University Hospital in line with the Declaration of Helsinki (1964).

- ***Inclusion for all participants*:** All participants spoke French as their native language. They did not present with previous neurological, psychiatric problems or history of severe brain injury (except brain abnormalities associated with AD or KS for patients). No participants presented with contraindications for an MRI scan (claustrophobia, pacemaker, foreign metallic object). For patients, clinical neuroimaging examinations ruled out other etiologies that could explain memory impairments.
- ***Inclusion criteria specific to KS:*** KS patients were recruited as inpatients at Caen University Hospital (*n* = 9) and from a nursing home (Maison Vauban, Roubaix, France; *n* = 9). They met the criteria for alcohol-induced major neurocognitive disorder, amnestic-confabulatory type, persistent (DSM-5 [2]). All patients presented with a history of chronic and heavy alcohol drinking, even though it was difficult to obtain an accurate quantification of their alcohol consumption because of amnesia. A multidisciplinary team of specialists examined each patient to ensure an accurate diagnosis of KS. All patients had a Mini-Mental State Examination (MMSE [13]) score ≥ 18.
- ***Inclusion criteria specific to AD*:** AD patients were recruited from the local memory clinic (Caen University Hospital) and in partnership with care facilities in the region of Normandy, France. All patients fulfilled the standard criteria for AD diagnosis reported by the National Institute of Neurological and Communicative Disorders and Stroke and the Alzheimer’s Disease and Related Disorders Association (NINCDS-ADRDA) [14]. Each patient was classified either in the MAD or SAD group based on the results of the MMSE. MAD group included AD patients with a MMSE score ≥ 18 to match the global cognitive deterioration of KS patients. To take account of AD progression, AD patients with an MMSE score < 18 were included in the SAD group.
- ***Inclusion criteria specific to HC*:** All HC had preserved performance on the MMSE for their age and education level. None consumed more than 3 standard drinks (2 for women) per day on a regular basis, as recommended by the World Health Organization.

As expected, all patient groups had lower Total MMSE score than HC, with KS and MAD presenting similar global cognitive deterioration and SAD exhibiting the most severe scores. On the Recall subtest of the MMSE, the three patient groups had significantly lower results than HC but performed similarly. For the Learning subtest, only the SAD group was impaired while for the Orientation and Attention subtests, KS and MAD performed lower than HC but better than SAD. On the Language subtest, the two AD groups performed lower than HC, with SAD presenting poorer performance than MAD. KS had preserved language results. On the Praxis subtest, only the MAD group had preserved results (Table 1). The SAD group had lower results than the KS and MAD groups.

### 2.2. Neuroimaging Examination

A high-resolution T1-weighted anatomical image was acquired for each participant on a Philips Achieva 3T scanner (Cyceron Imaging Center, Caen, France) using a 3D fast-field echo sequence (180 sagittal slices; thickness = 1 mm; repetition time = 20 ms; echo time = 4.6 ms; flip angle = 10°; field of view, 256 × 256 mm^2^; matrix, 256 × 256). Imaging data were preprocessed and analyzed using the SPM12 toolbox (www.fil.ion.ucl.ac.uk/spm (accessed on 25 April 2023)). The data were normalized to the Montreal Neurological Institute (MNI) template and segmented into gray matter (GM), white matter (WM), and cerebrospinal fluid (CSF). Normalized unmodulated images from controls were averaged with a threshold at 0.5 to create a binary gray matter mask for statistical analyses. For each participant, the total intracranial volume (TIV) was calculated based on the sum of the individual volumes of GM, WM, and CSF.

### 2.3. Statistical Analyses

**Figure 1:**
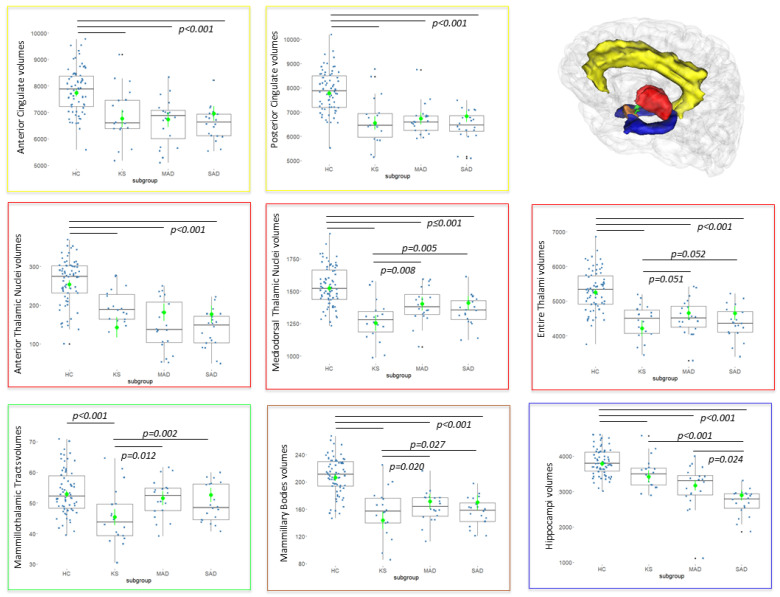
Volumetric comparisons between healthy controls (HC), patients with Korsakoff’s syndrome (KS), moderate Alzheimer disease (MAD) and severe Alzheimer disease (SAD) in several regions of the Papez circuit. In the upper right corner of the figure, regions of the Papez circuit considered in the analysis. Yellow: anterior and posterior cingulate gyri. Red: Thalamus. Blue: Hippocampus. Brown: Mammillary bodies. Green: Mammillothalamic tract. We used a generalized linear model (GLM): estimated marginal means are shown in green with vertical lines showing 95% Confidence Intervals. When F-tests were significant (*p* < 0.001, corrected for multiple comparisons), post-hoc tests were carried out (Tukey, unequal sample size). Statistically significant post-hoc tests between groups are shown with an overhead horizontal black line and associated *p*-value shown on the plots. Raw data are also illustrated via boxplots and volumes are expressed in mm^3^.

Volumes from regions belonging to the Papez circuit (Figure 1), and considered to be the hallmarks for amnesia, were extracted for further statistical analyses. The hippocampi, thalami as well as anterior and posterior cingulate gyri, were extracted from the Hammers’ Atlas of the medial temporal lobe [15]. The anterior thalamic nuclei, the mediodorsal nuclei and mammillothalamic tracts (MTT) were separated using the thalamic histological atlas [16]. Finally, the mammillary bodies were extracted from a single-subject brain atlas [17]. Modulated gray matter maps were used to extract the volumes for all the above regions, except for the MTT, for which modulated white matter maps were used. The volumes of these ROIs were compared across groups using a generalized linear model (GLM) with the group as the independent variable (four groups: HC, MAD, SAD, KS) and age and TIV as covariates. Post-hoc tests (Tukey’s test, unequal sample sizes) were conducted when appropriate.

## 3. Results

The GLM showed that, for all 8 regions, there was a significant group effect on the regional volumes (Hippocampi: F(3,122) = 47.3; Entire thalami: F(3,122) = 38.3; Anterior thalamic nuclei: F(3,122) = 38.28; Mediodorsal thalamic nuclei: F(3,122) = 30.44; Anterior cingulate gyrus: F(3,122) = 27.0; Posterior cingulate gyrus: F(3,122) = 50.6; Mammillary bodies: F(3,122) = 46.6; MTT F(3,122) = 9.78; *p* < 0.001 in all cases, corrected for multiple comparisons using the method of Bonferroni). 

Further post-hoc analyses showed that the volumes of the entire thalami were significantly smaller in all patient groups compared to HC (*p* < 0.001 in all cases). Shrinkage tended to be more severe in the KS group compared to both the MAD (*p* = 0.051) and SAD (*p* = 0.052) groups, which did not differ from each other (*p* = 1). For the mediodorsal thalamic nuclei and mammillary bodies, we found a similar pattern of significant results: smaller volumes in the KS group compared to both MAD (*p* = 0.008 and *p* = 0.02 respectively) and SAD (*p* = 0.005 and *p* = 0.027 respectively) groups.

The anterior thalamic nuclei, as well as the anterior and posterior cingulate gyri, were affected to the same extent in the three patient groups compared to HC (*p* < 0.001 in all cases). 

The volume of the hippocampi was significantly smaller in all patient groups compared to HC (*p* < 0.001 in all cases). Shrinkage was significantly more severe in the SAD group compared to both KS (*p* < 0.001) and MAD (*p* = 0.024) groups, which did not differ between each other. 

Finally, the volume of the MTT was lowest in the KS group and significantly different from HC (*p* < 0.001), MAD (*p* = 0.012) and SAD (*p* = 0.002), which did not differ significantly from each other. 

An outlier patient was observed in the regional volumetric measurements in the MAD group, whose value is even lower than those from the SAD group for certain regions. Statistical analyses were performed with and without this subject and all results remained the same, reaching significance, albeit different *p*-values. Since groups of SAD and MAD are selected based on established criterion, and there were no clinical reasons to exclude this patient, the data was kept in the analysis for the sake of completeness and integrity.

Results accounting for the two covariates are depicted in Figure 1 in terms of estimated marginal means and 95% intervals. Raw data are also presented in the boxplots. 

## 4. Discussion

In the existing literature, structural brain abnormalities observed in KS and AD are often reported in separate studies, providing little opportunity to directly compare these clinical populations. The novelty of the present study is to address this issue head-on. Data were collected from two independent cohorts of KS and AD patients at the same research site and using the same image acquisition protocols with the same MRI machine. Two groups of patients with different severities of AD were included to examine the potential effect of AD progression on the KS vs. AD comparison.

The present study indicates that several nodes of the Papez circuit were structurally damaged in both AD and KS, while the remaining gray matter nodes under study seemed to be differentially involved in the pathophysiological mechanisms underlying these diseases. The cingulate cortex, the hippocampus (at a moderate stage only) and the anterior thalamic nuclei were damaged to the same extent in AD and KS amnesia. 

Regarding the cingulate cortex, abnormalities have previously been observed in PET studies measuring cerebral glucose metabolism in patients with severe episodic memory deficits. Hypometabolism of the posterior cingulate cortex is classically found at an early stage of AD [18] and the cingulate cortex was shown as the only brain structure with hypometabolism in each of the 9 KS patients in a previous study [19]. Structural and functional abnormalities in the cingulate cortex are thus shared by patients with AD or KS and could be relevant to consider in the pathophysiology of these memory diseases [9,20]. 

The hippocampus is considered the predominant brain region responsible for episodic memory function [20] and remains the main focus of attention in AD research. In agreement with numerous previous investigations [21,22] and as expected, we found hippocampal shrinkage in the two groups of AD, with worsening of hippocampal atrophy with disease progression. This latter result emphasizes how crucial it is to consider disease trajectory when interpreting the extent of volume shrinkage measured in a degenerative disease such as AD. Our findings also support significant hippocampal damage in KS patients [10], even though it has not systematically been found in the literature [23] potentially due to imaging acquisition limitations. To our knowledge, only one study has directly compared regional brain volume deficits in 5 KS patients and 20 AD patients [10]. After controlling for age differences between groups, the authors found comparable hippocampal volume deficits, which related to memory impairments in both groups. We found similar results in the present study, which included a much larger group of KS as well as groups of MAD and KS matched for memory performance and global cognitive deterioration on a basic screening tool. 

Thalamic abnormalities are considered a cardinal feature of KS [24]. As expected, we found volume deficits of the anterior thalamus in KS patients compared with controls, in accordance with previous postmortem [6] and neuroimaging studies [25,26]. In agreement with previous studies conducted in AD [9,27,28], we also found thalamic shrinkage in both MAD and SAD patients. Previous work described thalamus shrinkage even in patients with amnestic Mild Cognitive Impairment [8,29,30,31], implicating thalamic atrophy at the early stages of the disease and in the pathophysiology of the associated episodic memory deficits. The thalamus seems especially vulnerable in prodromal AD, challenging the idea that it would only reflect a consequence of medial temporal lobe dysfunction [9]. This is in accordance with the historical description of neuropathology in the hippocampus and anterodorsal thalamus at the same stage of AD [3]. Thalamic abnormalities may thus directly contribute to the development of cognitive deficits in AD [32]. In theory, anterior thalamic changes would be associated with episodic memory deficits while mediodorsal thalamic changes could explain prodromal cases with predominantly executive deficits [9]. 

KS and AD thus share, within the Papez circuit, a pattern of regional brain shrinkage, which could reflect the involvement of some common pathways leading to amnesia, irrespective of the etiology. The direct comparison of thalamic abnormalities in AD and KS provides new evidence that confirms the contribution of the thalamus to amnesia, above KS, and its key role in episodic memory [33]. In patients with developmental amnesia [34], usually considered as a selective episodic memory disorder associated with hypoxia-induced hippocampal atrophy of early onset, a relationship was found between the patients’ thalamic volumes and their memory performance. When considering volumetric measurements in the Papez circuit, these data could question the relevance of the classic nosography between hippocampal and diencephalic amnesia. In agreement with this assumption, previous neuropsychological studies that compared episodic memory performance in AD and KS revealed a normal forgetting rate in the two patient groups suggesting, according to the author, an acquisition or learning deficit in both types of amnesia [35]. Similarly, contextual memory (temporal recency judgement in a recognition task) was affected to the same extent in AD and KS [36]. However, other studies that compared non-AD medial temporal lobe amnesia (patients with herpes encephalitis or hypoxia) and diencephalic amnesia (mainly including patients with KS) showed some differences in the patterns of impaired memory components. For example, concerning contextual memory, patients with diencephalic amnesia were impaired in retaining temporal information but had preserved performance for spatial one, while the opposite pattern of results was found in patients with medial temporal lobe amnesia [37]. The degenerative nature of AD may make it difficult to use data collected in this disease in the framework of the hippocampal versus diencephalic amnesia. 

Contrary to what we observed for the anterior thalamic nuclei, the shrinkage of the mediodorsal nuclei was more severe in KS than in the AD groups. Given the putative role of this thalamic nuclei in cognition [38], this finding is in accordance with the neuropsychological profile of KS and non-KS patients with alcohol use disorder, frequently characterized by attention, working memory and executive dysfunction [39]. Shrinkage of the mediodorsal nuclei does not seem related to amnesia and is shared by KS and alcohol use disorder patients [7]. In patients with severe AD, such cognitive deficits can also be observed but they may rather be related to neocortical damage.

Mammillary bodies were affected in the three patient groups but more severely in KS than in AD. Shrinkage of the mammillary bodies is consistently described in KS [23,26,40,41,42] but its specific contribution to the cognitive and brain pathophysiology remains unclear, mainly because it is difficult to study the impact of mammillary bodies abnormalities in isolation [5]. From an anatomical perspective, it is unlikely that mammillary body lesions explain KS amnesia since they do not affect the fornical afferents to the anterior thalamic nuclei nor the efferents from the anterior thalamic nuclei to the cingulate cortex. This leads to only a partial disconnection, which is contrary to lesions of the anterior thalamic nuclei that result in complete disconnection within the Papez circuit [5]. Mammillary body shrinkage has also been reported in AD with up to 25% of volume loss [43], but not in patients with Mild Cognitive Impairment [44]. Even in developmental amnesia, mammillary bodies shrinkage is frequent [34]. These findings reinforce the assumption that mammillary bodies abnormalities occur in patients with amnesia, potentially because of a disconnection or dysfunction in the Papez circuit, but do not initiate the pathophysiological mechanisms. The severity of the volume deficits found in KS compared with AD corroborates the hypothesis of a great vulnerability of this brain region to thiamine deficiency, leading to Wernicke’s encephalopathy and potentially to KS [5]. 

The mammillothalamic tracts (MTT) were observed as damaged in the KS group only, suggesting that its alterations could be specific to this pathology. The MTT effectively connects the mammillary bodies, which itself receives input from the hippocampus, to the anterior thalamic nuclei [45]. Lesions to the MTT bilaterally, accompanied by damage to the anterior thalamic nuclei, have been sufficient to cause acute and irreversible memory disorder that is very similar to KS [46]. Furthermore, another study [47] showed that 7 out of 12 patients with thalamic infarct who had damage to the MTT performed worse in the verbal episodic memory task than the 5 patients with preserved MTT. Taken together, these suggest that lesions to the anterior thalamic nuclei could be, to a certain extent, the result of a disconnection process through the MTT, having failed to receive part of the inputs from the hippocampus coming through the mammillary bodies [45,48].

This study finds its originality in its cohorts that are difficult to recruit and carefully select. The main limitation is the absence of a common and refined neuropsychological assessment. This absence is related to the fact that two patient groups come from different research programs conducted in the same imaging center, and thus sharing structural imaging modalities, but that included different cognitive evaluations. The common cognitive evaluation is limited to the MMSE, which makes it impossible to infer reliable and fine brain-behavior relationships. Our study provides the basis for developing a heuristic model that must be incremented by adding, in future studies, other imaging modalities, with a more detailed cognitive battery, to examine whether a similar pattern of hippocampal or anterior thalamic shrinkage observed in AD and KS is associated with similar or different profiles of alterations, including but not limited to, episodic memory components, structural and functional connectivity, metabolism, amyloid and tau deposition. 

A next step could also be to compare AD- versus alcohol-related minor neurocognitive disorders (patients with Mild Cognitive Impairments and patients with alcohol use disorder) across both cross-sectional and longitudinal studies to better understand the cascade of events governing the pathophysiological mechanisms that can potentially lead to AD and KS.

## 5. Conclusions

Our findings provide new direct evidence regarding the specificities and commonalities in the pattern of volume deficits within the Papez circuit in AD and KS, two major neurocognitive disorders leading to amnesia. Our study fits into the broader consideration of the substrates of amnesia and challenges the relevance of considering amnesia in reference to classic descriptions as being the result of critical damage to one non-overlapping region rather than the aftermath of a cascade of pathophysiological events within the Papez circuit. These data reinforce the relevance of examining not only the hippocampus or the thalamus, but a network of connected brain regions as the substrate of memory functioning [33]. From a clinical perspective, establishing the global or focal profiles of alterations will help to propose preventive or adaptive care to patients with amnesia.

## Figures and Tables

**Table 1:** Description of the population (mean ± standard deviation and range).

	HC N = 70	MAD Patients N = 20	SAD Patients N = 20	KS Patients N = 18	Statistics	Post-Hoc Comparisons
**Gender (men/women)**	24/46	8/12	2/18	8/10	X^2^ = 6.36; *p* = 0.09	n.a
**Age**	68.3 ± 10.1 (50–86)	78.7 ± 4.77 (70–88)	79.5 ± 5.78 (71–91)	55.6 ± 5.59 (44–67)	F = 35.1; *p* < 0.001	KS < HC < (MAD = SAD)
**Education**	10.1 ± 1.93 (6–17)	10.1 ± 3.15 (6–17)	9.05 ± 2.48 (6–15)	10.1 ± 2.37 (6–15)	F = 1.2; *p* = 0.314	n.a
**MMSE: Total Score** (Max. 30)	28.9 ± 1.10 (26–30)	22.6 ± 3.03 (18–27)	11.8 ± 3.56 (3–17)	23.2 ± 2.71 (18–27)	F = 309; *p* < 0.001	HC > (KS = MAD) > SAD
MMSE: Orientation * (Max. 10)	9.93 ± 0.315 (8–10)	7.50 ± 1.46 (5–10)	2.45 ± 2.04 (0–8)	7.83 ± 1.72 (4–10)	F = 204; *p* < 0.001	HC > (KS = MAD) > SAD
MMSE: Learning * (Max. 3)	3 ± 0.0 (3–3)	2.94 ± 0.250 (2–3)	2.40 ± 0.883 (0–3)	2.89 ± 0.471 (1–3)	F = 11.4; *p* < 0.001	(HC = KS = MAD) > SAD
MMSE: Recall * (Max. 3)	2.60 ± 0.715 (0–3)	0.267 ± 0.458 (0–1)	0.150 ± 0.489 (0–2)	0.667 ± 0.767 (0–2)	F = 118; *p* < 0.001	HC > (KS = MAD = SAD)
MMSE: Attention * (Max. 5)	4.85 ± 0.357 (4–5)	3.87 ± 1.77 (0–5)	0.70 ± 1.17 (0–4)	3.33± 1.41 (0–5)	F = 94.4; *p* < 0.001	HC > (KS = MAD) > SAD
MMSE: Language * (Max. 8)	7.56 ± 0.608 (5–8)	7.07 ± 0.704 (6–8)	5.85 ± 1.42 (1–8)	7.50 ± 0.707 (6–8)	F = 22.8; *p* < 0.001	HC = KS; HC > MAD > SAD; (KS = MAD) > SAD
MMSE: Praxis * (Max. 1)	0.971 ± 0.170 (0–1)	0.733± 0.458 (0–1)	0.300 ± 0.470 (0–1)	0.722 ± 0.461 (0–1)	F = 22.0; *p* < 0.001	HC = MAD; KS = MAD; HC > KS > SAD; MAD > SAD

HC: healthy controls; MAD: moderate Alzheimer’s disease; SAD: severe Alzheimer’s disease; KS: Korsakoff syndrome; MMSE: Mini-Mental State Examination. Post-hoc tests: Tukey HSD (unequal N); n.a = not applicable; * MMSE subscores were missing for 2 HC and 4 MAD patient.

## Data Availability

De-identified data supporting the findings of the study can be made available upon request from the corresponding author.
